# Viscoelastic Properties of Polymeric Microneedles Determined by Micromanipulation Measurements and Mathematical Modelling

**DOI:** 10.3390/ma16051769

**Published:** 2023-02-21

**Authors:** Zhihua Zhang, Guangsheng Du, Xun Sun, Zhibing Zhang

**Affiliations:** 1 School of Chemical Engineering, University of Birmingham, Birmingham B15 2TT, UK; 2 Changzhou Institute of Advanced Manufacturing Technology, Changzhou 213164, China; 3 West China School of Pharmacy, Sichuan University, Chengdu 610041, China

**Keywords:** single microneedles, micromanipulation, mechanical strength, viscoelastic analysis

## Abstract

Microneedles, including dissolvable ones made from biocompatible and biodegradable materials, have been widely studied and can potentially be used for transdermal drug delivery, disease diagnosis (sampling), skin care, etc. Characterizing their mechanical properties is essential, as being mechanically strong enough to pierce the skin barrier is one of the most fundamental and crucial requirements for them. The micromanipulation technique was based on compressing single microparticles between two flat surfaces to obtain force and displacement data simultaneously. Two mathematical models had already been developed to calculate the rupture stress and apparent Young’s modulus, which can identify variations of these parameters in single microneedles within a microneedle patch. In this study, a new model has been developed to determine the viscoelasticity of single microneedles made of hyaluronic acid (HA) with a molecular weight of 300 kDa loaded with lidocaine by using the micromanipulation technique to gather experimental data. The modelling results from the micromanipulation measurements suggest that the microneedles were viscoelastic and their mechanical behaviour was strain-rate dependent, which implies that the penetration efficiency of viscoelastic microneedles can be improved by increasing their piercing speed into the skin.

## 1. Introduction

Microneedles are needle-like structures with a length shorter than 1000 μm and a tip width of several tens of microns, and usually, an array of microneedles (e.g., 10 × 10) are fabricated in a patch to be used. They are designed to effectively penetrate the stratum corneum (the topmost layer of skin) without touching the blood vessels or nerves, thus generating no pain and having a low infection risk in contrast with traditional hypodermic needle sticks [1]. They can be used for transdermal drug delivery [2], disease diagnosis (sampling) [3], skin care [4], etc. Microneedles can be classified into several types based on different criteria ranging, from pyramid-shaped, truncated cone-shaped, and other complicated shaped microneedles based on their shapes [2], to glass, metal, and polymer microneedles based on the materials they are made from [5], and to solid, coated, dissolvable (dissolving), and hollow microneedles based on their structure and solubility in body liquid [6]. Dissolvable microneedles made from biocompatible and biodegradable materials that can dissolve or degrade in the human body after administration can potentially have a wide range of applications, including sustained and controlled drug and vaccine delivery and skin care because of their advantages, including no residue of sharp waste after administration and good safety [1,7,8,9]. For instance, sustained release of a loaded drug is easy to achieve by modifying the dissolvable microneedles with cross-linkable groups [10].

Microneedles should have desirable fundamental properties, including chemical, structural, geometrical, thermal, and mechanical, to function properly in end-use applications. Their chemical compositions usually determine their solubility, stability, drug loading capacity, etc. [11]. Thermal properties (glass transition temperature and melting point) can affect storage stability and mechanical strength under different temperature conditions. The mechanical strengths are usually affected by the chemical composition, structure, and geometry of the microneedles [12]. Being mechanically strong enough to pierce the skin barrier is one of the most fundamental and crucial requirements for microneedles. However, some types of microneedles, e.g., dissolvable ones, are mechanically weaker in comparison with those made of metal and glass [5,8]. Therefore, precisely characterising their mechanical strength is essential to ensure their penetration capacity in academic research and new product development based on microneedles.

Several techniques have been used to test the mechanical strength of microneedles, indirectly or directly. In indirect measurement, the mechanical strength of microneedles is estimated by testing macro samples prepared using the same material rather than by directly testing the microneedles [13]. However, the results obtained using this method can have great uncertainty because the fabrication mechanism of the microneedles is not the same as that of the macro samples used in tensile tests, which can result in different mechanical property results.

To directly determine their mechanical strength, microneedles on a patch were usually tested together, and the average force of single microneedles was calculated by dividing the whole loading force by the number of microneedles on the patch [7,9,14,15,16,17,18]. However, the possible variations of mechanical properties among single microneedles across the patch, which are very important to ensure their reproducible penetration efficiency, cannot be identified. Moreover, in most of these studies, the average failure force was used to characterise the mechanical strength of microneedles and to predict their penetration efficiency by comparing it with the insertion force required to pierce the skin with microneedles with the same geometric parameters, especially the tip size, as the required insertion force was mainly determined by the interfacial area at the needle tip and the corresponding pressure [14,16]. Therefore, in order to carry out the comparison, the required insertion force of microneedles with the same or similar geometry needs to be determined first. Otherwise, it is not sufficient to draw conclusions simply using the failure force without knowing the geometrical and structural parameters of the tested microneedles, as the failure force is not an intrinsic mechanical property for a given intrinsic mechanical strength [8].

Atomic Force Microscopy (AFM) was used to determine the (relative) indentation hardness of single microneedles by indenting their tips. Their penetration capacity was predicted by comparing the slope of the force-displacement curves of the microneedles to that of an artificial skin tested with the same indentation probe [19]. However, the obtained hardness was a local value corresponding to where the nanoindentation was applied, and rupture parameters cannot be obtained because of the limited applied force that AFM can generate. Similar problems remain in other techniques for characterising the mechanical strength of single particles, including pressure probing [20], micropipette aspiration [21], and optical/magnetic tweezers [22], and thus they are not proper for the determination of the mechanical strength of microneedles, whose failure forces are usually in the range of milli-Newtons [8].

In our previous studies [8,10], the micromanipulation technique, which had been widely used to determine the mechanical strength of single microparticles, was used to test the mechanical strength of single hyaluronic acid (HA) based microneedles. Two mathematical models were developed to determine their mechanical strength and Young’s modulus. The obtained mechanical strength parameters, including the rupture force, displacement at rupture, deformation at rupture, rupture stress, and Young’s modulus, were thoroughly compared, and two major results were found, i.e., the loading of the drug significantly decreased the mechanical strength of microneedles, and a higher molecular weight of HA resulted in a greater Young’s modulus [8]. Moreover, the rupture stress was found to be intrinsic and better to be used to characterise the mechanical strength of microneedles than the rupture force. With the two developed mathematical models, the intrinsic mechanical property parameters as well as their variations for single microneedles can be determined, which can be used to predict their penetration capacity comprehensively and reliably even without knowing their geometrical or structural information. However, it was found that the penetration efficiency of microneedles by impact insertion (high speed) was much higher than by manual insertion (low speed) [23], which implies that the mechanical behaviours of microneedles can vary with the insertion procedure, including the insertion speed, i.e., the microneedles show some speed-dependent behaviour. Therefore, new research needs to be carried out on their viscoelastic and/or viscoplastic behaviour.

In this paper, the elastic model for single microneedles developed in our previous study [8] has been extended to determine their viscoelasticity by accounting for the time-dependent behaviours of single microneedles from the experimental data of the micromanipulation tests. HA microneedles loaded with lidocaine have been tested using the micromanipulation technique, and the obtained force versus time/displacement data have been analysed using the developed viscoelastic model to obtain their intrinsic viscoelastic property parameters.

## 2. Materials and Methods

### 2.1. HA Microneedles Loaded with Lidocaine

Microneedles with the shape of a truncated pyramid made from HA and a molecular weight of 300 kDa loaded with lidocaine were used in this study. They were fabricated by micro moulding, and the detailed preparation procedure is reported elsewhere [8]. Briefly, polydimethylsiloxane (PDMS) moulds were first duplicated from a stainless-steel mould and the microneedle body parts were formed by compressing the HA solution (200 mg/mL) dissolved with lidocaine into the PDMS mould using pressured air. After being dried in an anhydrous silica gel environment (room temperature and humidity) for 0.5 h, the microneedle base plate was prepared by adding pure hyaluronic acid (HA) to the PDMS mould. Finally, the microneedle patches were peeled off from the PDMS mould after drying in an anhydrous silica gel environment (room temperature and humidity) for another 4 h. Their formula is illustrated in Table 1.

### 2.2. Micromanipulation of Single Microneedles

**Figure 1:**
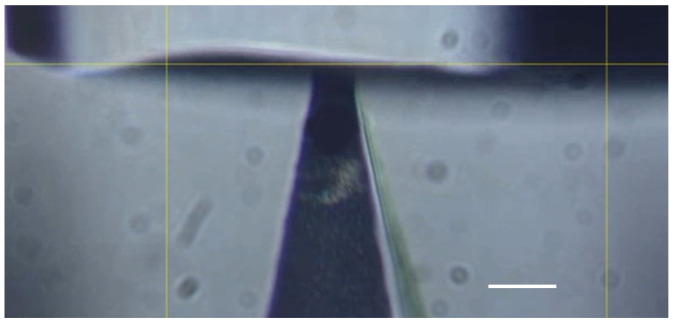
A Lido-HA300kDa microneedle under compression and holding (tip width: 11.6 μm, scale bar: 20 μm).

The micromanipulation technique is based on the diametrical compression of single microparticles/microneedles between two parallel flat surfaces to different deformations, including rupture, during which force-displacement/time data are obtained simultaneously. The detailed principle has been reported elsewhere [24,25,26,27]. In the micromanipulation tests, the bottom of a microneedle patch was fixed to the surface of a glass slide using double-sided tape (30401, Deli Group Co., Ltd., Ningbo, China). The glass slide was fixed onto the sample stage of the micromanipulation rig. Single microneedles of Lido-HA300kDa were compressed to a certain deformation and held for 10 s under the transducer probe, as illustrated in Figure 1. The ambient temperature and humidity were 17 ± 2 °C and 38 ± 3% Rh, respectively.

In order to assess whether the double-sided tape can generate any viscoelastic behaviour in the system, a small glass with a similar dimension (6.5 mm × 6.5 mm) to the microneedles’ base was placed on top of the double-sided tape, which had a thickness of ~ 0.1 mm (see Figure S1 in the supplementary information). The diameter of the probe was around 60 µm. The compression displacement of the force transducer probe on the small glass slide was set to be 8–30 μm, and the corresponding force versus sampling time was recorded.

### 2.3. Modelling of the Viscoelastic Behaviour of Single Microneedles

#### 2.3.1. Derivation of the Viscoelastic Model

**Figure 2:**
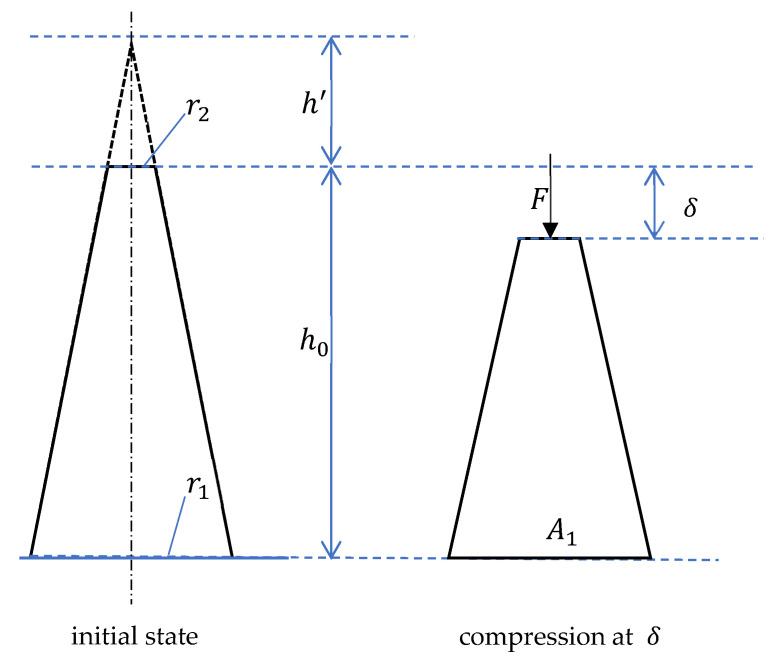
Schematic diagram of a single microneedle under compression. $A_{1}$ is the area of the microneedle bottom, $h_{0}$ is the initial length of the microneedle body, $r_{1}$ and $r_{2}$ are the initial half side length of a quadrangular microneedle base and tip respectively, and $h^{\prime }$ is the height of the missing tip.

The schematic diagram of a single microneedle under compression is illustrated in Figure 2. Assuming the microneedle is perfectly sharp initially, the relationship between the force *F* and displacement *δ* of single microneedles under compression within the elastic limit can be expressed using the elastic model in Equation (1) developed in the previous study [8].

(1) $$F=\frac{A_{1}h^{\prime }E}{\left(h_{0}+h^{\prime }\right)h_{0}}\delta$$

where E is Young’s modulus of the microneedle.

From similarity,

(2) $$\frac{h^{\prime }}{h_{0}+h^{\prime }}=\frac{r_{2}}{r_{1}}$$

$A_{1}$ can be calculated using the following equation for the microneedles studied in this research with a shape of a truncated pyramid:

(3) $$A_{1}=4r_{1}^{2}$$

Combination of Equations (1)–(3) leads to

(4) $$F=\frac{4r_{1}r_{2}E}{h_{0}}\delta$$

The mathematical relationship between Young’s modulus E and the shear modulus G can be expressed using Equation (5) [28].

(5) $$G=\frac{E}{2\left(1+\nu \right)}$$

where ν is the Poisson’s ratio. Letting

(6) $$n_{1}=\frac{4r_{1}r_{2}}{h_{0}}$$

and combining Equations (4)–(6) leads to the following equation:

(7) $$F=\left(1+\nu \right)\cdot 2G\cdot n_{1}\delta$$

Following the approach of [28], replacing the constant term 2G with the time-varying shear modulus G(t) leads to

(8) $$F\left(t\right)=\left(1+\nu \right)\cdot G\left(t\right)\cdot n_{1}\delta$$

Viscoelasticity can be seen as a combination of a viscos unit and a linear elastic unit, which are most commonly described by a Newtonian dashpot and a Hookean spring, respectively [29]. Several models can be used to describe the viscoelastic behaviour of materials, and the Maxwell and Kelvin models are the two most basic ones, which can be seen as the serial and parallel combination of the viscos and linear elastic units. Other complex viscoelastic models can be built from different combinations of Maxwell and Kelvin models [26,29]. The generalised Maxwell model (also called the Prony series [28]), which is built by the combination of n terms of the basic Maxwell model, is more general and semi-empirical [26]. It has been successfully used to describe the spherical indentation and relaxation of soft biological tissues [30] and the compression and relaxation of agarose micro-particles [28]. Thus, it is also used in this paper to model the relaxation of microneedles, which are made from biocompatible materials. During the relaxation, the time-dependent shear modulus can be expressed using the following equation [28]:

(9) $$G\left(t\right)=C_{0}+\overset{n}{\underset{i=1}{\sum }}C_{i}e^{-\frac{t}{\tau_{i}}}$$

Similarly, the corresponding force can be expressed as [28].

(10) $$F\left(t\right)=B_{0}+\overset{n}{\underset{i=1}{\sum }}B_{i}e^{-\frac{t}{\tau_{i}}}$$

where $\tau_{i},\;i=1,\;2,\;\ldots ,\;n$ is the relaxation time. The relationship between the coefficients $C_{0}$, $C_{i}$ and $B_{0}$, $B_{i}$ are

(11) $$B_{0}=\left(1+\nu \right)\cdot n_{1}\delta_{0}\cdot C_{0}$$

and

(12) $$B_{i}=\left(1+\nu \right)\cdot n_{1}\delta_{0}\cdot \Phi_{i}\cdot C_{i}$$

where $\delta_{0}$ is the maximum displacement corresponding to the end of loading, and $\Phi_{i}$ is the ramp correction factor given by [28]

(13) $$\Phi_{i}=\frac{\tau_{i}}{t_{R}}\left(e^{t_{R}/\tau_{i}}-1\right)$$

where $t_{R}$ is the rising time from the onset of loading to the onset of relaxation.

It is worth pointing out that the value of the relaxation times $\tau_{1},\;\tau_{2},\;\cdots$ indicate how fast each relaxation term takes place and the magnitudes of $B_{1},\;B_{2},\;\cdots$ suggest the significance of each relaxation term. Smaller values of relaxation times mean faster relaxation, and bigger values of magnitudes have a more significant influence on overall relaxation.

The instantaneous shear modulus $G_{0}$ and long-term shear modulus $G_{\infty }$ can then be estimated from the coefficients $C_{0}$, $C_{1}$, $C_{2}$, …, $C_{n}$ [28].

(14) $$G_{0}=\frac{\sum_{i=0}^{n}C_{i}}{2}$$

and

(15) $$G_{\infty }=\frac{C_{0}}{2}$$

The corresponding instantaneous Young’s modulus, $E_{0}$, and long-term Young’s modulus, $E_{\infty }$, can be calculated by [28].

(16) $$E_{0}=2\left(1+\nu \right)G_{0}$$

and

(17) $$E_{\infty }=2\left(1+\nu \right)G_{\infty }$$

#### 2.3.2. Determination of Viscoelastic Parameters Based on Numerical Optimization

The viscoelastic model with degree n≥2 as shown in Equation (10) cannot be used analytically to determine the model parameters from experimental data, but the viscoelastic parameters can be obtained by numerical optimization to minimize the sum of the squared difference between the predicted force ${\hat{F}}_{i}$ and the experimental force $F_{i}$ based on the following equation:

(18) $$\underset{B_{0},\;\;B_{1},\cdots \;B_{n},\tau_{1},\cdots ,\;\tau_{n}}{\min}\overset{N}{\underset{i=1}{\sum }}{\left({\hat{F}}_{i}-F_{i}\right)}^{2}$$

where the predicted force, ${\hat{F}}_{i}$, is calculated using Equation (10) and N is the number of data points during the holding experiment.

After the determination of $B_{0}$, $B_{1}$, …, $B_{n}$, $\tau_{1}$, $\tau_{2}$, …, $\tau_{n}$, the coefficients $C_{0}$, $C_{1}$, $C_{2}$, …, $C_{n}$, and the ramp correction factors $\Phi_{1}$, $\Phi_{2}$, …, $\Phi_{n}$ are determined from which the instantaneous and long-term shear and Young’s moduli $G_{0}$, $G_{\infty }$, $E_{0}$, and $E_{\infty }$ can be obtained.

#### 2.3.3. Prediction of the Loading Force Using the Viscoelastic Parameters

With the parameters obtained from the force relaxation data [30], the following equation can be used to predict the loading force data:

(19) $$F\left(t\right)=\left(1+\nu \right)\cdot n_{1}\cdot \int_{0}^{t}G\left(t-u\right)\cdot \left\{\frac{d}{du}\left[\delta \left(u\right)\right]\right\}du$$

If the compliance of the force transducer is negligible, δ(t) can be expressed as [28]

(20) δ(t)=Vt

where V is the moving speed of the micromanipulator holding the force transducer. Substituting Equations (9) and (20) into Equation (19) leads to the following equation:

(21) $$F\left(t\right)=\left(1+\nu \right)n_{1}V\left(C_{0}t+\overset{n}{\underset{i=1}{\sum }}C_{i}\tau_{i}(1-e^{-\frac{t}{\tau_{i}}}\right)$$

which can be used to predict the force during the compression of single microneedles if the compliance of the force transducer is negligible.

However, if the compliance of the force transducer is not negligible relative to the imposed displacements, significant errors can result from using Equation (21). Here, two strategies can be used. One is using the average moving speed of the force transducer tip, which can be determined from the corresponding displacement versus time data, to substitute the moving speed of the micromanipulator V. Another is using numerical solutions. The displacement of the force transducer tip δ(t) is given by [27]

(22) δ(t)=Vt−cF(t)

where c is the compliance of the force transducer. Substituting Equation (22) into Equation (19) results in

(23) $$\begin{array}{ll} F\left(t\right)= & \left(1+\nu \right)n_{1}\int_{0}^{t}G\left(t-u\right)[\frac{d}{du}\left(Vu\right)]du\\ & -\left(1+\nu \right)n_{1}c\int_{0}^{t}G\left(t-u\right)\frac{d}{du}\left[F\left(u\right)\right]du \end{array}$$

The second part, which includes F(t) itself, cannot be solved mathematically. To deal with this problem, a numerical solution can be used by decomposing the loading history into a sum of infinitesimal loading steps based on the Boltzmann superposition principle [31]

(24) $$F\left(t\right)=\left(1+\nu \right)n_{1}\overset{\infty }{\underset{i=1}{\sum }}G\left(t-\Delta t_{i}\right)\left[\delta \left(t\right)-\delta \left(t-\Delta t_{i}\right)\right]$$

where, $\Delta t_{i},\;i=1,\;2,\cdots$, is the infinitesimal time interval in which step loading is applied.

In practice, the acquisition time ($t_{s})$ can be used as $\Delta t_{i},\;i=1,\;2,\cdots$ to decompose the loading history, then Equation (24) can be written as follows:

(25) $$F\left(jt_{s}\right)=\left(1+\nu \right)n_{1}\overset{j}{\underset{i=1}{\sum }}G\left(it_{s}-t_{s}\right)\left[\delta \left(it_{s}\right)-\delta \left(it_{s}-t_{s}\right)\right]$$

where j is the sampling number. Equation (25) can also be written as

(26) $$F_{j}=\left(1+\nu \right)n_{1}\overset{j}{\underset{i=1}{\sum }}G_{i-1}\left(\delta_{i}-\delta_{i-1}\right)$$

and

(27) $$F_{j-1}=\left(1+\nu \right)n_{1}\overset{j-1}{\underset{i=1}{\sum }}G_{i-1}\left(\delta_{i}-\delta_{i-1}\right)$$

from which the following equation can be derived

(28) $$F_{j}-F_{j-1}=\left(1+\nu \right)n_{1}G_{j-1}\left(\delta_{j}-\delta_{j-1}\right)$$

The corresponding discrete form of Equation (22) can be written as

(29) $$\delta_{j}=jVt_{s}-cF_{j}$$

and

(30) $$\delta_{j-1}=\left(j-1\right)Vt_{s}-cF_{j-1}$$

Thus

(31) $$\delta_{j}-\delta_{j-1}=Vt_{s}-cF_{j}+cF_{j-1}$$

A combination of Equation (28) and Equation (31) leads to the following recursive equation

(32) $$F_{j}=\{\begin{array}{c} \;0,\;\;j=1\\ \;\\ \frac{\left(1+\nu \right)n_{1}Vt_{s}G_{j-1}+\left(1+n_{1}cG_{j-1}\right)F_{j-1}}{1+\left(1+\nu \right)n_{1}cG_{j-1}},\;\;j=2,\;3,\;4,\;\ldots \end{array}$$

and

(33) $$G_{j-1}=C_{0}+\overset{n}{\underset{i=1}{\sum }}C_{i}e^{\frac{t_{j-1}}{\tau_{i}}},\;\;j=2,\;3,\;4,\ldots$$

Equations (32) and (33) can be used to predict the forces during loading in the typical loading-holding experiment of microneedle tests in a numerical recursive way without the restriction of negligible force transducer compliance as required when Equation (21) is used.

## 3. Results and Discussion

### 3.1. Force-Time Data of Lido-HA300kDa Microneedles under Compression and Holding

**Figure 3:**
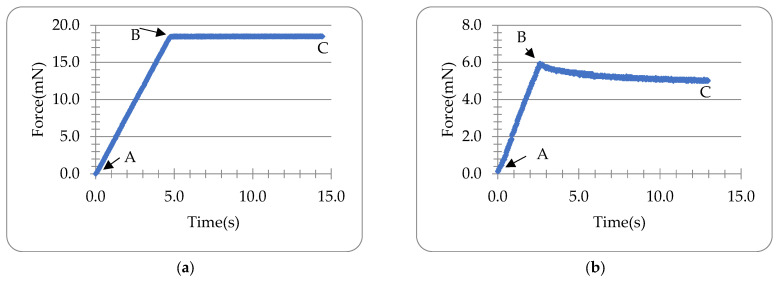
Typical force-time curves of the loading-holding test of the glass slide (**a**) and Lido-HA300kDa microneedles (**b**) (tip width $2r_{2}=$ 16.1 μm, bottom width $2r_{1}=250\;$μm, $h_{0}=700$ μm) whose bases were placed on the double-sided tape. A: Onset of compression. B: Onset of holding (end of compression). C: End of holding. Compression speed was 2 µm/s.

Typical curves of force versus time data during compression and holding of the glass slide (as control) and single Lido-HA300kDa microneedles whose bases were placed on the double-sided tape are illustrated in Figure 3a and Figure 3b, respectively. Clearly, the force increased during compression in both cases. During the holding of the glass slide (which is considered to be very rigid) on the tape, there was no significant force relaxation, whereas the force dropped notably during the holding of the microneedle, which suggests that some viscoelastic behaviour of the microneedle existed.

### 3.2. Elastic Analysis

**Figure 4:**
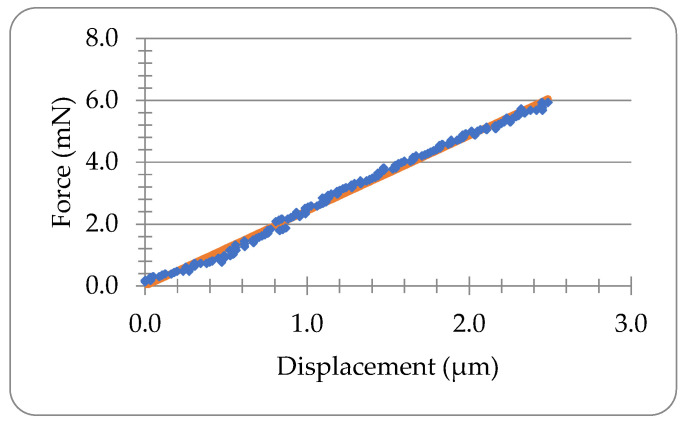
Elastic analysis of the microneedle in Figure 3b. The line presents the fitting using Equation (4).

If the viscos behaviour is neglected, the force-displacement data of the Lido-HA300kDa microneedle during compression can be fitted into the elastic model in Equation (4) as illustrated in Figure 4. The coefficient of determination (CoD) is 0.99, which suggests good fitting. The Youngs modulus obtained is 422.8 MPa.

### 3.3. Viscoelastic Analysis

**Figure 5:**
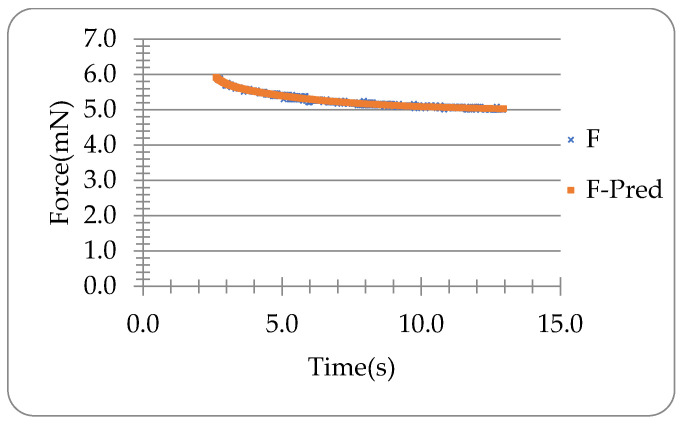
Experimental force (F) and predicted force (F-Pred) versus time during holding.

Letting the degree n=2 and fitting the force-time data during the holding of the microneedle in Figure 3b into Equation (10) optimised with Equation (18) using Microsoft^®^ Excel Solver, the coefficients $B_{0}$, $B_{1}$, $B_{2}$ and the time constants $\tau_{1}$ and $\tau_{2}$ can be obtained as shown in Table 2. It can be seen that the viscoelastic model with two relaxation times can predict the experimental holding data of the microneedle in Figure 3b well (CoD=0.98), as illustrated in Figure 5. The rising time $t_{R}=2.60\;$s, the displacement up to the end of compression $\delta_{0}=2.52$ μm, and the ramp correction factors $\Phi_{1}$ and $\Phi_{2}$ are 1.43 and 384.88, respectively. The coefficients $C_{0}$, $C_{1}$, $C_{2}$ are shown in Table 3 obtained using Equations (11) and (12).

The relaxation time of the second relaxation (0.32 s) is nearly one order smaller than the loading time (2.60 s), which indicates the relaxation is quite rapid. Although the first relaxation time (3.82 s) is in the same order as the loading time (2.60 s), the magnitude of the first relaxation ($B_{1}=1.55\;$mN) is much smaller than the second one ($B_{2}=534.35\;$mN), which suggests that the second relaxation is dominant for the relaxation of the tested microneedle in Figure 3b.

The instantaneous shear modulus $G_{0}$, long-term shear modulus $G_{\infty }$, instantaneous Young’s modulus $E_{0}$ and the long-term Young’s modulus $E_{\infty }$ of the microneedle in Figure 3b have been obtained using Equations (14)–(17), and the values are shown in Table 4. The Poisson’s ratio ν of 0.5 was used. In last section, the Youngs modulus value without considering the viscos behavior is 422.8 MPa, which is expected to be between the values of the instantaneous Young’s modulus (513.3 MPa) and long-term Young’s modulus (343.2 MPa). Other tested Lido-HA300kDa microneedles yielded comparable results.

**Figure 6:**
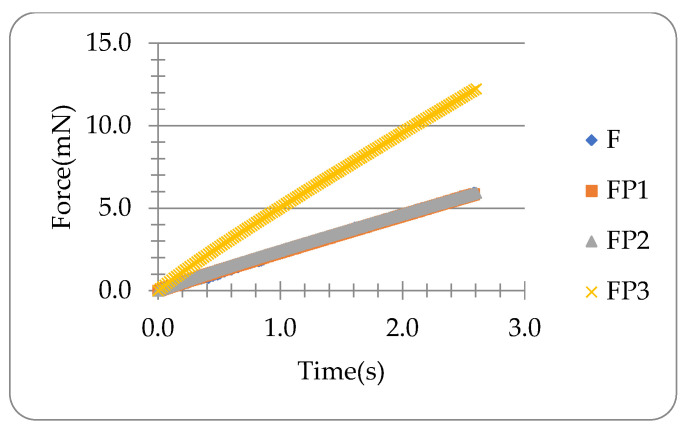
Predicted force data in comparison with the experimental data corresponding to compression of the microneedle. F is the experimental force. F-P1 is predicted by the numerical method using Equation (32). F-P2 is predicted using Equation (21) with the average moving speed of the force transducer tip (=$\delta_{0}/t_{R}$). F-P3 is obtained using Equation (21) with the pre-set moving speed of the micromanipulator holding the force transducer.

The force data during loading for the microneedle in Figure 3b have been predicted using the parameters obtained from the holding (i.e., force relaxation) data, which are shown in Figure 6. It can be seen that the results obtained from the approach with the average moving speed of the force transducer tip (F-P2) and the numerical approach (FP-1) are both consistent with the experimental data, and thus they both can be used to predict the compression force utilising the viscoelastic parameters obtained from the data of the force relaxation corresponding to the holding. The predicted force F-P3 deviates from the experimental data significantly as the compliance of the transducer c is 0.5 μm/mN, which is not negligible with such a big force in the order of mN or above.

Totally, twenty-six single Lido-HA300kDa microneedles were tested. Their experimental data during compression were analysed using the elastic model by neglecting the viscos behaviour and the obtained mean Young’s modulus value was 496.5 ± 31.6 MPa, which lies between the obtained mean instantaneous and long-term Young’s modulus values (619.0 ± 37.2 MPa and 390.5 ± 24.1 MPa, respectively) obtained from the analysis of their holding data using the developed viscoelastic model. The variations of the viscoelastic property parameters, including the instantaneous and long-term Young’s moduli, can mainly result from the physiochemical properties and composition of the polymer matrix used to prepare the microneedles, which has been verified in [8], where variations of the mechanical strength over a patch of the four tested microneedle samples made from HA with and without loaded drugs were identified. It was reported that the value of Young’s modulus of human skin also depends on the loading speed and direction as well as the test methods, which were found to be 5–100 kPa by indentation tests, 0.025–140 MPa by tensile and torsion tests, and 25–260 kPa by suction tests [32]. The instantaneous and long-term Young’s modulus values of Lido-HA300kDa microneedles are significantly greater than those of human skin, and therefore they could be strong enough to reliably pierce it.

Moreover, the difference between the instantaneous and long-term moduli is quite significant, implying that different penetration efficiency can be obtained by adjusting the insertion speed, i.e., with a higher insertion speed, more efficient penetration can be achieved because the apparent Young’s modulus value should increase with compression speed.

## 4. Further Discussion

To precisely determine the intrinsic mechanical property of single microneedles, the micromanipulation technique had been used, and an elastic model has been developed in our previous studies [8] to determine Young’s modulus of single microneedles from the experimental data obtained at a given compression speed, which may be considered as the apparent Young’s modulus. Young’s modulus is an intrinsic mechanical property of microneedles that are independent of their geometrical or structural information. However, the microneedles made from dissolvable polymers usually show some speed-dependent (viscoelastic) behaviour, and their mechanical properties, including the apparent Young’s modulus, can vary with the strain rate used to test them. Therefore, in this paper, the elastic model has been extended to analyse the viscoelastic behaviours of microneedles, and a mathematical model has been developed to determine their viscoelastic parameters, including the instantaneous and long-term Young’s moduli. The apparent Young’s modulus value obtained from using the purely elastic model [8] is a value at a certain compression speed (i.e., 2 μm/s), whereas the instantaneous and long-term Young’s moduli obtained from using the viscoelastic model are the upper and lower limits of Young’s modulus values, which correspond to compressions at infinitely large and infinitely small speeds, respectively. Results of Lido+HA300kDa microneedles show that their Young’s modulus values at a compression speed of 2 μm/s (obtained from the elastic model) were between their instantaneous and long-term Young’s modulus values, and the difference between the latter two is quite significant, which suggests that the microneedles show higher stiffness when they penetrate the skin with an increasing insertion speed, and consequently a higher penetration efficiency can be obtained. Although it was found that drug loading could decrease the mechanical strength of microneedles [8,10], reliable piercing can still be achieved by increasing the insertion speed using a mechanical insertion device, such as an impact-insertion applicator [23] for microneedles loaded with drugs.

## 5. Conclusions

In conclusion, a viscoelastic model has been developed in this paper to determine the viscoelastic parameters of single microneedles tested using the micromanipulation technique. In combination with the rupture strength model and elastic model developed in the previous study [8], the intrinsic mechanical property parameters as well as their variations in single microneedles can be precisely determined from the micromanipulation tests, which can be used to predict their penetration capacity comprehensively and reliably even without knowing their geometrical or structural information. A numerical recursive equation (Equation (32)) has been developed to predict the loading force using the viscoelastic parameters obtained from the holding data, which has the advantage that the compliance of the force transducer is included in comparison with the analytical method in which the compliance was negligible [28].

Moreover, the three mathematical models can also be used to analyse the experimental data of microneedle arrays tested using other techniques, including the displacement-force test station [15] and the micro-mechanical test machine with a microforce sensor [18]. Therefore, they can find wide applications in academic research and the development of new products based on microneedles.

## Figures and Tables

**Table 1:** HA microneedles loading with lidocaine.

Abbreviation	Matrix
Lido-HA300kDa	Lidocaine + Hyaluronic acid (MW: 300,000 Da) Lidocaine to HA weight ratio 4:5

**Table 2:** Calculated values of the coefficients and relaxation times of the microneedle in Figure 3b.

Width (μm)	$B_{0}$ (mN)	$B_{1}$ (mN)	$B_{2}$ (mN)	$\tau_{1}$ (s)	$\tau_{2}$ (s)
16.1	4.98	1.55	534.35	3.82	0.32

**Table 3:** Values of coefficients $C_{0}$, $C_{1}$, $C_{2}$.

$C_{0}$ (MPa)	$C_{1}$ (MPa)	$C_{2}$ (MPa)
228.8	49.6	63.8

**Table 4:** Values of shear moduli and Youngs moduli.

$G_{0}$ (MPa)	$G_{\infty }$ (MPa)	$E_{0}$ (MPa)	$E_{\infty }$ (MPa)
171.1	114.4	513.3	343.2

## Data Availability

Not applicable.

## Supplementary Materials

The following supporting information can be downloaded at: https://www.mdpi.com/article/10.3390/ma16051769/s1, Figure S1: Loading-holding test of the glass slide whose base was placed on the double-sided tape.

## Nomenclature

$A_{1}$	area of the microneedle bottom (m^2^)
$B_{0}$	relaxed force (Equation (10)) (N)
$B_{i},\;i=1,\;2,\;\cdots$	proportional constants (Equation (10)) (N)
c	compliance of the force transducer (m/N)
$C_{0}$	$\mathrm{is}\;\mathrm{related}\;\mathrm{to}\;B_{0}$
$C_{i},\;i=1,2,\;\cdots$	proportional constants (Equation (9)) (N)
CoD	coefficient of determination
E	Young’s modulus (Pa)
$E_{0}$	instantaneous Young’s modulus (Pa)
$E_{\infty }$	long-term Young’s modulus (Pa)
${\hat{F}}_{i}$	predicted force from models (N)
$F_{i}$	the i^th^ force (N)
F(t)	time-dependent force (N)
G	shear modulus (Pa)
$G_{0}$	instantaneous shear modulus (Pa)
$G_{\infty }$	long-term shear modulus (Pa)
G(t)	time-dependent shear modulus (Pa)
$h_{0}$	height of the microneedle (m)
$h^{\prime }$	height of the missing tip assuming the microneedle is perfectly sharp (m)
n	degree number in the viscoelastic analysis
N	number of data points during the holding
$r_{1}$, $r_{2}$	half side length of a quadrangular microneedle base and tip (m)
t	time (s)
$t_{R}$	rising time (time taken to compress a microneedle) (s)
$t_{s}$	acquisition time (sampling time) (s)
V	compression speed (m/s)
Greek Symbols	
δ	displacement (m)
$\delta_{0}$	maximum displacement before relaxation (m)
$\tau_{i},\;i=1,\;2,\;\cdots$	relaxation times (s)
$\Phi_{i},\;i=1,\;2,\;\cdots$	ramp correction factors (Equation (13))
ν	Poisson’s ratio
