# Supplementary material for: Viscoelastic Properties of Polymeric Microneedles Determined by Micromanipulation Measurements and Mathematical Modelling
Source: Materials (Basel). 2023 Feb 21;16(5):1769. doi: 10.3390/ma16051769 (PMC10003889; doi:10.3390/ma16051769)
Supplement: Supplementary file 1 [file materials-16-01769-s001.zip › materials-2158516-SI.pdf]

Supplementary Information

Viscoelastic Properties of Polymeric Microneedles Determined by Micromanipulation Measurements and Mathematical Modelling

Zhihua Zhang [1,2], Guangsheng Du [3], Xun Sun [3] and Zhibing Zhang [1,*]

[1] School of Chemical Engineering, University of Birmingham, Birmingham B15 2TT, UK
[2] Changzhou Institute of Advanced Manufacturing Technology, Changzhou 213164, China
[3] West China School of Pharmacy, Sichuan University, Chengdu 610041, China
* Correspondence: z.zhang@bham.ac.uk

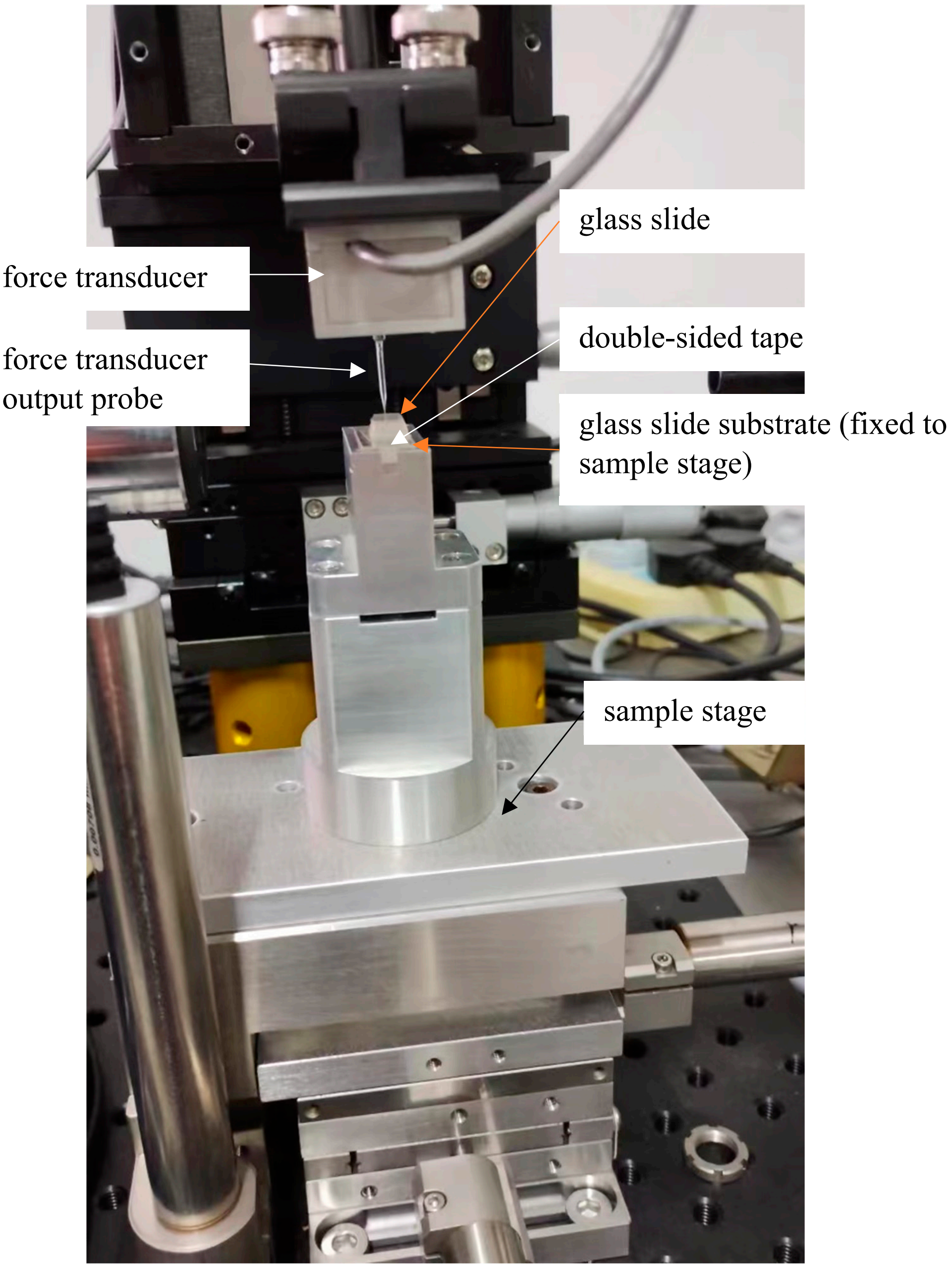

Figure S1. Loading-holding test of the glass slide whose base was placed on the double-sided tape. In order to assess whether the double-sided tape can generate any viscoelastic behaviour in the system, a small glass with a similar dimension (6.5mm ×6.5mm) to the microneedles' base was placed on top of the double-sided tape, which had a thickness of ~0.1 mm. The diameter of the probe was around 60 µm. The compression displacement of the force transducer probe on the small glass slide was set to be 8-30 µm, and the corresponding force versus sample time was recorded
